# The impact of demographic, clinical, genetic, and imaging variables on tau PET status

**DOI:** 10.1007/s00259-020-05099-w

**Published:** 2020-11-19

**Authors:** Rik Ossenkoppele, Antoine Leuzy, Hanna Cho, Carole H. Sudre, Olof Strandberg, Ruben Smith, Sebastian Palmqvist, Niklas Mattsson-Carlgren, Tomas Olsson, Jonas Jögi, Erik Stormrud, Young Hoon Ryu, Jae Yong Choi, Adam L. Boxer, Maria L. Gorno-Tempini, Bruce L. Miller, David Soleimani-Meigooni, Leonardo Iaccarino, Renaud La Joie, Edilio Borroni, Gregory Klein, Michael J. Pontecorvo, Michael D. Devous, Sylvia Villeneuve, Chul Hyoung Lyoo, Gil D. Rabinovici, Oskar Hansson, Alzheimer’s Disease Neuroimaging Initiative, PREVENT-AD research group

**Affiliations:** 1 Clinical Memory Research Unit, Lund University, Lund, Sweden; 2 Alzheimer Center Amsterdam, Department of Neurology, Amsterdam Neuroscience, Amsterdam UMC, VU University Medical Center, Vrije Universiteit Amsterdam, Amsterdam, The Netherlands; 3 Department of Neurology, Gangnam Severance Hospital, Yonsei University College of Medicine, Seoul, South Korea; 4 School of Biomedical Engineering and Imaging Sciences, King’s College London, London, UK; 5 Dementia Research Centre, Department of Neurodegenerative Disease, UCL Institute of Neurology, London, UK; 6 Centre for Medical Image Computing, Department of Medical Physics, University College London, London, UK; 7 Department of Neurology, Skåne University Hospital, Lund, Sweden; 8 Wallenberg Centre for Molecular Medicine, Lund University, Lund, Sweden; 9 Department of Radiation Physics, Skåne University Hospital, Lund, Sweden; 10 Department of Clinical Physiology and Nuclear Medicine, Skåne University Hospital, Lund, Sweden; 11 Memory Clinic, Skåne University Hospital, Malmö, Sweden; 12 Department of Nuclear Medicine, Gangnam Severance Hospital, Yonsei University College of Medicine, Seoul, South Korea; 13 Division of applied RI, Korea Institute Radiological and Medical Sciences, Seoul, South Korea; 14 Department of Neurology, Memory and Aging Center, University of California San Francisco, San Francisco, USA; 15 F. Hoffmann-La Roche Ltd, Basel, Switzerland; 16 Avid Radiopharmaceuticals, Philadelphia, PA, USA; 17 Departments of Psychiatry and Neurology & Neurosurgery, Douglas Mental Health University Institute, McGill University, Montreal, Quebec, Canada; 18 Department of Radiology and Biomedical Imaging, University of California San Francisco, San Francisco, USA; 19 Molecular Biophysics and Integrated Bioimaging Division, Lawrence Berkeley National Laboratory, Berkeley, CA, USA

**Keywords:** PET, Tau, Aβ, Alzheimer’s disease, MCI, Dementia

## Abstract

**Purpose:**

A substantial proportion of amyloid-β (Aβ)+patients with clinically diagnosed Alzheimer’s disease (AD) dementia and mild cognitive impairment (MCI) are tau PET–negative, while some clinically diagnosed non-AD neurodegenerative disorder (non-AD) patients or cognitively unimpaired (CU) subjects are tau PET–positive. We investigated which demographic, clinical, genetic, and imaging variables contributed to tau PET status.

**Methods:**

We included 2338 participants (430 Aβ+ AD dementia, 381 Aβ+ MCI, 370 non-AD, and 1157 CU) who underwent [^18^F]flortaucipir (*n* = 1944) or [^18^F]RO948 (*n* = 719) PET. Tau PET positivity was determined in the entorhinal cortex, temporal meta-ROI, and Braak V-VI regions using previously established cutoffs. We performed bivariate binary logistic regression models with tau PET status (positive/negative) as dependent variable and age, sex, *APOEε*4, Aβ status (only in CU and non-AD analyses), MMSE, global white matter hyperintensities (WMH), and AD-signature cortical thickness as predictors. Additionally, we performed multivariable binary logistic regression models to account for all other predictors in the same model. Results Tau PET positivity in the temporal meta-ROI was 88.6% for AD dementia, 46.5% for MCI, 9.5% for non-AD, and 6.1% for CU. Among Aβ+ participants with AD dementia and MCI, lower age, MMSE score, and AD-signature cortical thickness showed the strongest associations with tau PET positivity. In non-AD and CU participants, presence of Aβ was the strongest predictor of a positive tau PET scan.

**Conclusion:**

We identified several demographic, clinical, and neurobiological factors that are important to explain the variance in tau PET retention observed across the AD pathological continuum, non-AD neurodegenerative disorders, and cognitively unimpaired persons.

## Introduction

Tau accumulation is a key neuropathological feature of Alzheimer’s disease (AD) and is closely linked to synaptic loss, neurodegeneration, and cognitive deficits [1]. The advent of positron emission tomography (PET) ligands with high affinity for the tau aggregates formed in AD now enables the visualization and quantification of tau pathology in vivo [2]. Recently, one of these tau PET tracers ([^18^F]flortaucipir) was approved by the US Food and Drug Administration to support the diagnostic process in patients with suspected AD dementia [3]. This is an important step towards the clinical application of tau PET.

However, several hurdles need to be overcome in order to accelerate the transition of tau PET from an investigational technique to a diagnostic biomarker. One of these challenges is the observation of negative tau PET scans in individuals suspected of having symptomatic AD [4–7] or, conversely, positive tau PET scans in individuals suspected for a non-AD neurodegenerative disorder like frontotemporal lobar degeneration (behavioral or language phenotype) or Parkinsonian disorders [5, 6, 8, 9]. This can potentially hamper the interpretation of the tau PET result or lead to suboptimal patient selection for a clinical tau PET scan. A better understanding of factors that contribute to tau PET status could alleviate this concern.

Based on previous literature [4, 7, 8, 10–23] and data availability, we evaluated in this large multicenter study the impact of demographic (age and sex), clinical (Mini-Mental State Examination [MMSE]), genetic (apolipoprotein [*APOE*] genotype), and imaging (cortical thickness, white matter hyperintensities [WMH], and amyloid-β [Aβ] status) variables on tau PET status across persons with AD dementia, mild cognitive impairment (MCI), and non-AD neurodegenerative disorders, and cognitively unimpaired (CU) individuals.

## Material and methods

### Participants

We included 2338 participants from the Memory Disorder Clinic of Gangnam Severance Hospital (Seoul, South Korea, *n* = 310) and the Swedish BioFINDER 1 (*n* = 228) and 2 (*n* = 719) studies at Lund University (Lund, Sweden), the University of California San Francisco (UCSF) AD Research Center (San Francisco, USA, *n* = 201), the PREVENT-AD study (*n* = 134), the Alzheimer’s disease neuroimaging initiative (ADNI, *n* = 655), and Avid Radiopharmaceuticals studies (A04 [*n* = 36], A05 [*n* = 219], A08 [*n* = 76], and the placebo arm of the Eli Lilly solanezumab Expedition-3 study [*n* = 85]). Of these participants, 1695 (72.5%) underwent [^18^F]flortaucipir PET and 643 (27.5%) [^18^F]RO948 PET. According to the NIA-AA diagnostic criteria [24, 25] and a research framework [26], we only included Aβ+ AD dementia (*n* = 430) and MCI (*n* = 381) patients (determined using PET and/or CSF, see previous work for details [5, 6]). A total of 370 participants were diagnosed with a non-AD neurodegenerative disorder according to formal diagnostic criteria [5, 6], including Parkinson’s disease (*n* = 123), progressive supranuclear palsy (*n* = 58), dementia with Lewy bodies (*n* = 51), behavioral variant frontotemporal dementia (*n* = 50), corticobasal syndrome (*n* = 27), the semantic (*n* = 19) and non-fluent (*n* = 17) variants of primary progressive aphasia, multiple system atrophy (*n* = 12), and dementia not otherwise specified (*n* = 14). CU individuals (*n* = 1157) performed within normal limits on neuropsychological testing and had no significant neurological or psychiatric illnesses. In addition to tau PET, all participants underwent a medical history and neurological examination, MRI, and neuropsychological testing. Written informed consent was obtained from all participants, and local institutional review boards for human research approved the study. The study was performed in accordance with the ethical standards as laid down in the 1964 Declaration of Helsinki and its later amendments or comparable ethical standards.

### Acquisition of PET and MRI data

PET images were acquired using a Biograph mCT PET/CT scanner (Siemens Medical Solutions) in Seoul [11]; Discovery and Discovery MI PET scanners (GE medical systems) in BioFINDER 1 and 2, respectively [5, 6]; a Biograph 6 Truepoint PET/CT scanner (Siemens Medical Solutions) for UCSF patients [18], a Siemens High-Resolution Research Tomograph (HRRT) scanner in PREVENT-AD [15], and across multiple scanners in the multi-center ADNI [27] and Avid Radiopharmaceuticals [7] cohorts. All PET data were locally reconstructed into 4 × 5-min frames for the 80–100-min ([^18^F]flortaucipir) and 70–90-min ([^18^F]RO948) intervals post-injection. MR images were acquired on a 3.0T Discovery MR750 scanner (GE medical systems) in Seoul [11], 3.0T Tim Trio or Skyra scanner (Siemens Medical Solutions) in BioFINDER [5, 6], a 3.0T Tim Trio or Prisma scanner (Siemens Medical Solutions) at UCSF [18], a 3.0 Tim Trio scanner (Siemens Medical Solutions) in PREVENT-AD [15], and across multiple scanners in the multi-center ADNI [27] and Avid Radiopharmaceuticals [7] cohorts.

### T1-weighted MRI processing

MRI data were centrally processed (at Lund University), using previously reported procedures [5, 6]. Briefly, cortical reconstruction and volumetric segmentation were performed with the FreeSurfer (v6.0) image analysis pipelines (http://surfer.nmr.mgh.harvard.edu/). The MP-RAGE images underwent correction for intensity homogeneity [28], removal of non-brain tissue [29], and segmentation into gray matter (GM) and white matter (WM) with intensity gradient and connectivity among voxels [30]. Cortical thickness was measured as the distance from the GM/WM boundary to the corresponding pial surface [31]. Reconstructed data sets were visually inspected for accuracy, and segmentation errors were corrected. We computed “AD-signature” cortical thickness [32] comprising bilateral entorhinal, inferior and middle temporal, and fusiform cortex regions of interest (ROI).

### [^18^F]flortaucipir PET processing

PET images were first re-sampled to obtain the same image size (128 × 128 × 63 matrix) and voxel dimensions (2.0 × 2.0 × 2.0 mm) across centers. Next, PET images were centrally processed (at Lund University) using previously reported procedures [5, 6]. [^18^F]Flortaucipir images were motion-corrected using AFNI’s 3dvolreg, time-averaged and rigidly co-registered to the skull-stripped MRI scan. Voxelwise standardized uptake value ratio (SUVR) images were created using inferior cerebellar gray matter as the reference region [33]. FreeSurfer (v6.0) parcellation of the T1-weighted MRI scan was applied to the PET data transformed to subjects’ native T1 space to extract mean regional SUVR values. In line with our previous work [5, 6], we calculated the mean [^18^F]flortaucipir and [^18^F]RO948 SUVR in the entorhinal cortex (early tau region), a temporal meta-ROI [32] comprised of a weighted average of entorhinal, amygdala, parahippocampal, fusiform, and inferior and middle temporal ROIs (intermediate tau region), and Braak stage V/VI encompassing widespread neocortical ROIs (late tau region). As tau PET positivity can be established using a variety of methods, we aimed to be consistent with our earlier work and use previously published and validated procedures for both tracers [5, 6]. Hence, we determined tau PET positivity in each of the aforementioned regions using the mean of elderly (69.1 ± 9.5 years) CU subjects + (2 * SD) for [^18^F]flortaucipir [6] and the mean of young (30.5 ± 6.6 years) Aβ-negative CU subjects + (2.5 * SD) [^18^F]RO948 [5]. This resulted in cutoffs of 1.39 (entorhinal cortex), 1.34 (temporal meta-ROI), and 1.28 (Braak V-VI) SUVR for [^18^F]flortaucipir, and 1.48 (entorhinal cortex), 1.36 (temporal meta-ROI), and 1.35 (Braak V-VI) SUVR for [^18^F]RO948.

### FLAIR MRI processing

T2-weighted fluid attenuated inversion recovery (FLAIR) images were available for 1660/2338 (71.0%) participants. We estimated total WMH volumes following a segmentation method described elsewhere [34]. Briefly, this method builds a Bayesian probabilistic data model based on a Gaussian mixture model with an evolving number of components. Due to distribution skewness, data were log-transformed prior to statistical analysis.

### Statistical analyses

To identify factors associated with tau PET positivity, we performed bivariate binary logistic regression models with tau PET status (positive/negative) in the three preselected regions (i.e., entorhinal cortex, temporal meta-ROI, and Braak V-VI) as dependent variable, and age, sex, *APOE ε*4 status, Aβ status (only in analyses including participants with a non-AD neurodegenerative disorder and CU individuals), MMSE, total WMH volumes (adjusted for intracranial volume), and AD-signature cortical thickness as predictors. Values for AD-signature cortical thickness and total WMH were divided by 10 and 1000 respectively, to obtain odds ratios in a comparable range to the other variables. Additionally, we performed multivariable binary logistic regression models to account for all other predictors in the same model. We excluded total WMH from the multivariable analysis due to a significant proportion (29%) of missing data. Finally, we conducted a post hoc analyses to estimate at which MMSE score the tau PET scan would be robustly positive in Aβ-positive participants with AD dementia and MCI. Using a non-parametric regression method (i.e., locally estimated scatterplot smoothing [35]), we computed the MMSE score at which the tau PET thresholds diverged from the 95% confidence interval of the slope reflecting tau PET SUVR vs MMSE scores. Significance level was set at 2-sided *P* < 0.05. We used R v3.5.2 for the statistical analyses.

## Results

### Participants

**Fig. 1:**
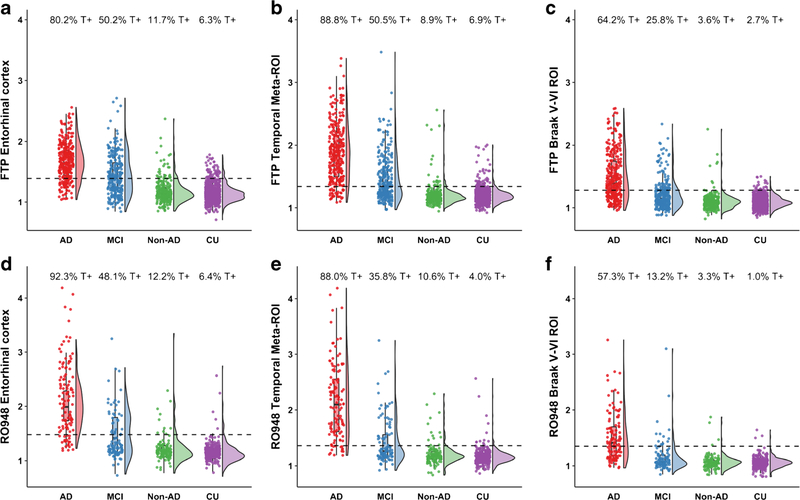
The *y*-axis represents the SUVR values for [^18^F]flortaucipir (FTP, panel **a**–**c**) and [^18^F]RO948 (panel **d**–**f**) for the entorhinal cortex (**a** and **d**), temporal meta-ROI (**b** and **e**), and Braak V/VI ROI for Aβ+ Alzheimer’s disease (AD) dementia, Aβ+ MCI, non-AD neurodegenerative disorders, and cognitively unimpaired (CU) participants. The percentages on top of each panel represent the proportion of tau PET–positive cases based on previously established cutoffs

The participant characteristics across diagnostic groups are presented in Table 1. Overall, the study participants were 69.4 ± 10.2 years old and 53.2% were males. By definition, all AD dementia and MCI patients were Aβ positive, and 65.5% (AD dementia) and 61.8% (MCI) carried at least one *APOE ε*4 allele. In the non-AD neurodegenerative disorder and CU group, 29.0% and 34.3% were Aβ positive and 32.5% and 35.7% *APOE ε*4 positive, respectively. Figure 1 shows the distribution of tau PET SUVR values and the positivity rates across the three ROIs, stratified by tau PET tracer. In the temporal meta-ROI, tau PET positivity was observed in 1.5% of Aβ-negative CU, 2.2% of Aβ-negative non-AD, 14.7% of Aβ-positive CU, 27.7% of Aβ-positive non-AD, 46.5% of Aβ-positive MCI, and 88.6% of Aβ-positive AD dementia.

### Tau PET positivity in amyloid-β+ AD dementia

Bivariate and multivariable binary logistic regression models in patients with Aβ+ AD dementia are presented in Table 2. The multivariable analysis indicated that younger age and lower MMSE scores were associated with higher rates of tau positivity across all ROIs. In addition, lower AD-signature cortical thickness was associated with higher rates of tau positivity in the temporal meta-ROI and the Braak V/VI ROI, and the presence of an *APOE ε*4 allele was associated with a higher rate of tau positivity in the temporal meta-ROI only. There were no significant associations between sex and tau PET positivity in both bivariate and multivariable models. WMH volumes were excluded from the multivariable analysis due to missing data, but bivariate models revealed no significant associations between global WMH volumes and tau PET positivity.

### Tau PET positivity in amyloid-β+ MCI

Bivariate and multivariable binary logistic regression models in patients with Aβ+ MCI are presented in Table 3. The multivariable analysis indicated that lower MMSE scores and lower AD-signature cortical thickness were associated with higher rates of tau positivity across all ROIs. In addition, presence of an *APOE ε*4 allele was associated with higher rates of tau positivity in the entorhinal cortex and temporal meta-ROI, and lower age was associated with a higher rate of tau positivity in the Braak V/VI ROI. There were no significant associations between sex and tau PET positivity in the multivariable models, but male sex was associated with a higher rate of tau positivity in the entorhinal cortex in the bivariate model. WMH volumes were excluded from the multivariable analysis due to missing data. The bivariate analysis indicated a significant association between global WMH volumes and tau PET positivity in the entorhinal cortex, but not in the temporal meta-ROI or the Braak V/VI ROI.

**Fig. 2:**
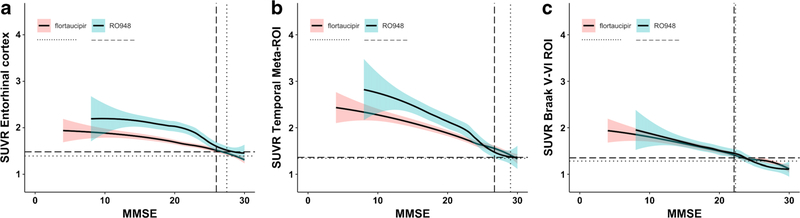
The graphs show non-linear associations between tau PET uptake and MMSE scores for both [^18^F]flortaucipir and [^18^F]RO948. The horizontal lines (dotted for [^18^F]flortaucipir and dashed for [^18^F]RO948) indicate previously established cutoffs for tau PET positivity, while the vertical lines indicate the MMSE score at which the 95% confidence interval of the slope no longer overlaps with the respective tau PET cutoff for each tracer

Since a lower MMSE score was consistently associated with higher rates of tau PET positivity across all ROI for both Aβ+ MCI and AD dementia, we performed a post hoc analysis to estimate around which MMSE score the tau PET is robustly positive (Fig. 2). This analysis indicated that the 95% confidence interval did not overlap with the tau PET positivity threshold at an MMSE of 27.6 ([^18^F]flortaucipir) or 26.0 ([^18^F]RO948) for the entorhinal cortex, 29.1 ([^18^F]flortaucipir) or 26.6 ([^18^F]RO948) for the temporal meta-ROI, and 22.2 ([^18^F]flortaucipir) or 21.9 ([^18^F]RO948) for the Braak V/VI ROI.

### Tau PET positivity in non-AD neurodegenerative disorders

Bivariate and multivariable binary logistic regression models in patients with a clinically diagnosed non-AD neurodegenerative disorder are presented in Table 4. The bivariate analysis indicated that most variables (except for sex) were associated with higher rates of tau positivity, especially in the entorhinal cortex and temporal meta-ROI, with Aβ positivity consistently showing the strongest associations. In the multivariable analysis, only Aβ positivity and lower MMSE scores remained significantly associated with higher rates of tau positivity in the entorhinal cortex and temporal meta-ROI. There were only 13 tau PET–positive cases in the Braak V-VI ROI (12 of them being Aβ positive), warranting cautious interpretation of these results.

### Tau PET positivity in cognitively unimpaired individuals

Bivariate and multivariable binary logistic regression models in CU participants are presented in Table 5. The bivariate analysis indicated that most variables (except for sex) were associated with higher rates of tau positivity, especially in the entorhinal cortex and temporal meta-ROI, with Aβ positivity consistently showing the strongest associations. In the multivariable analysis with tau PET positivity in the entorhinal cortex as the dependent variable, older age, presence of an *APOE ε*4 allele, Aβ positivity, lower MMSE, and lower AD-signature cortical thickness were associated with higher rates of tau positivity. For the temporal meta-ROI, significant associations were found for older age, male sex, and Aβ positivity. For the Braak V/VI ROI, significant associations were found for male sex only.

## Discussion

In this large multicenter study, we investigated how demographic (age and sex), clinical (MMSE), genetic (*APOE* genotype), and imaging/CSF (AD-signature cortical thickness, global WMH volumes, and Aβ status) variables were associated with tau PET status defined using previously established quantitative thresholds for [^18^F]flortaucipir and [^18^F]RO948. In Aβ-positive AD dementia and MCI, younger age, lower MMSE scores, and lower AD-signature cortical thickness showed the strongest associations with tau PET positivity. In non-AD neurodegenerative disorders and CU participants, the presence of Aβ was the strongest predictor of a positive tau PET scan. We thus identified several demographic, clinical, and neurobiological factors that are important to explain the variance in tau PET retention observed across the AD pathological continuum, non-AD neurodegenerative disorders, and CU persons.

Among Aβ-positive participants clinically diagnosed with AD dementia, the main predictors of a negative tau PET scan were older age and a higher MMSE score. At older age, a lower tau burden might be sufficient to produce a dementia syndrome due to the co-occurrence of other age-related pathologies (e.g., TDP-43, *α*-synuclein, or vascular pathology, [36, 37]) that contribute to cognitive decline (i.e., the “double-hit hypothesis” [38]) or to reduced efficiency of functional repair mechanisms associated with chronological aging that may magnify the cognitive consequences of tau pathology [16, 39]. Alternatively, in a proportion of participants, their Aβ positivity may be comorbid to a primary non-AD neurodegenerative disorder [40, 41], which would explain the negative tau PET scan because [^18^F]flortaucipir and [^18^F]RO948 seem to primarily bind to “AD-like” tau pathology and less to the tau aggregates formed in other tauopathies [42, 43]. A third explanation is the possibility of a selection bias, as older participants with multiple pathologies and other vulnerabilities who additionally have a high tau burden would become too cognitively impaired to participate in clinical research. The greater rates of tau PET–negative status with higher MMSE scores may be explained by the restricted sensitivity of both tau PET tracers [5, 6] that requires a significant pathological tau burden in order to cross the quantitative threshold [44], or by an inherent limitation of PET that requires a certain density of the target (Bmax) to allow unambiguous detection. This finding is in line with the drop in tau PET positivity rates in for example the temporal meta-ROI from AD dementia (88% for both tracers) to MCI (51% for [^18^F]flortaucipir and 36% for [^18^F]RO948). More modest associations with tau PET positivity were found for *APOE ε*4 status (temporal meta-ROI only) and lower AD-signature cortical thickness (temporal meta-ROI and Braak V-VI regions), and sex and global WMH volumes did not impact tau PET status in Aβ-positive AD dementia.

In Aβ-positive MCI, the main predictors for tau PET positivity were lower MMSE scores and decreased AD-signature cortical thickness. Both could be considered markers of disease progression, and in line with the findings in AD dementia and previous literature, there is a tight link between the amount of tau pathology and the level of neurodegeneration and cognitive impairment [11, 19, 22, 45–48]. To a greater degree than in AD dementia, *APOE ε*4 carriership was associated with a positive tau PET scan in the entorhinal cortex and temporal meta-ROI (but not in the Braak V/VI ROI), which is in accordance with earlier reports of a temporal lobe predilection of *APOE ε*4 [21, 49]. Furthermore, age played a less prominent role at the MCI stage of AD, as there was only a significant association with (younger) age and tau PET positivity in the Braak V/VI ROI. Sex and global WMH volumes did not impact tau PET status in Aβ-positive MCI.

In participants with a non-AD neurodegenerative disorder, positive Aβ status and lower MMSE scores were the only significant predictors of tau PET positivity in the multivariable models. The finding of positive Aβ status can be explained twofold. First, it could be due to a clinical misdiagnosis with AD as the primary pathological substrate for their dementia syndrome [41, 50]. This would fit the observation of (lower) MMSE as second predictor of tau PET positivity, as in more advanced disease stages, it may become more difficult to disentangle the symptomatic and radiological features across neurodegenerative disorder and thus increase their clinical and neuroanatomical overlap. Second, Aβ and tau pathology could be present as secondary pathology, whereas the clinical syndrome is driven by non-AD pathologies, as in aging populations where multiple proteinopathies can emerge in tandem [37]. It should be noted that in bivariate models, nearly all variables (except for sex) were associated with tau PET positivity, with the directionality of these associations always in line with AD risk factors (i.e., older age, *APOE ε*4 positivity, lower MMSE, reduced AD-signature cortical thickness, and elevated global white matter hyperintensity volumes), but these associations did not survive in multivariable models accounting for Aβ status. Finally, results for Braak V/VI regions should be interpreted with caution, as only 13 participants were tau PET positive in this group.

In CU individuals, positive Aβ status and older age were the strongest predictors of tau PET positivity in the entorhinal cortex and temporal meta-ROI. This is in line with an extensive literature on the interplay between Aβ and tau pathology in aging populations, suggesting that amyloid-β facilitates the neocortical spread of tau pathology [51]. In addition, there is evidence for a (possibly Aβ-independent) effect of advancing age on tau accumulation in the medial temporal lobe, which would explain why older age was only associated with tau PET positivity in the entorhinal cortex and temporal meta-ROI, but not in the Braak V-VI ROI. The multivariable model for the Braak V-VI ROI indicated male sex as the only significant predictor of tau PET status, but this finding should be interpreted with caution because this association did not reach significance in the bivariate model and the number of tau PET–positive CU participants in this ROI was limited. Similar to the results for non-AD neurodegenerative disorders, the bivariate models indicated that more “AD-like” characteristics of the predictors (e.g., *APOE ε*4 positivity, lower MMSE scores, and reduced AD-signature cortical thickness) were associated with tau PET positivity.

The main strengths of this study include the large sample size, the wide variety of clinical diagnoses, and the use of predefined cutoffs for tau PET positivity. There are also several limitations. First, inherent to the retrospective multicenter study design, harmonization between cohorts is complicated. For the imaging data, we attempted to minimize variability by analyzing all data centrally using an identical pipeline, but we acknowledge that differences in data acquisition and pre-processing remain. There likely also exists heterogeneity in the administration of the MMSE and in determining Aβ status using PET or CSF, the former likely being more conservative. Second, due to lack of statistical power to perform the analyses in individual non-AD neurodegenerative disorders, we combined them into a single diagnostic group. It is possible that the study outcomes would be different when focusing on specific non-AD neurodegenerative disorders. Third, the number of CU individuals (*n* = 26, 2.3%) and participants with non-AD neurodegenerative disorders (*n* = 13, 3.5%) who were tau PET positive in the Braak V/VI ROI was low. In addition to early (entorhinal cortex) and intermediate (temporal meta-ROI) tau regions, we aimed to also examine a more advanced tau region (i.e., Braak V/VI), but these results should be interpreted with caution. Fourth, we used two different tau PET tracers in this study and defined tau PET positivity using different albeit predefined methods. Based on a previous head-to-head comparison between [^18^F]flortaucipir and [^18^F]RO948 that demonstrated comparable neocortical tracer retention [52], we deemed it appropriate to combine them in the analyses. Fifth, some of the non-AD tauopathies included in this study (e.g., Progressive supranuclear palsy (PSP) or Corticobasal degeneration (CBD)) are characterized by specific isoforms of tau (i.e., 4R in case of PSP and CBD) that are not sufficiently detectable using [^18^F]flortaucipir or [^18^F]RO948 PET [5, 6]. With “tau PET positivity” in this study, we refer to the detection of “AD-like” tau aggregates, and we acknowledge that tau PET negativity does not exclude the presence of certain tau aggregates typically observed in other tauopathies.

The recent US Food and Drug Administration approval for use of [^18^F]flortaucipir PET to support the diagnostic process in patients with suspected AD dementia represents an important step towards application of tau PET in the clinic. The current study identified several demographic, clinical, and neurobiological factors that help explain the variance of tau PET retention observed across the AD pathological continuum, non-AD neurodegenerative disorders, and CU persons. This information might be incorporated into decision trees tailored to optimize patient selection for a clinical tau PET scan and to facilitate the interpretation of tau PET images. Future studies should focus on further development of visual read metrics [44], whether and how a quantitative threshold approach would add to a visual read, how threshold approaches for different tau PET tracers relate to each other and whether a standardized quantification approach would be feasible, and which cutoff in which ROI provides optimal diagnostic performance. This would be especially important to increase the sensitivity of tau PET tracers to detect tau pathology in early disease stages, while maintaining its excellent specificity.

## Figures and Tables

**Table 1:** Participant characteristics

	Aβ+ AD dementia	Aβ+ mild cognitive impairment	Non-AD neurodegenerative disorders	Cognitively unimpaired
N	430	381	370	1157
Age	71.5 (8.9)	71.6 (8.0)	69.4 (8.1)	67.9 (11.8)
Sex (% male)	55.6	46.5	53.5	55.3
*APOE* ε4 (% positive)	65.5	61.8	32.5	35.7
Amyloid-β (% positive)	100	100	29.0	34.3
MMSE	20.8 (4.5)	27.0 (2.4)	24.2 (5.5)	29.0 (1.3)
[^18^F]flortaucipir/[^18^F]RO948 (n)	313/117	275/106	247/123	860/297
AD-signature cortical thickness, mm	2.41 (0.19)	2.60 (0.20)	2.57 (0.23)	2.74 (0.14)
Global WMH volumes, log mm^3^	3.71 (0.48)	3.68 (0.48)	3.62 (0.44)	3.48 (0.45)


Data are presented as mean (standard deviation), unless otherwise stated

*AD* Alzheimer’s disease, *APOE* apolipoprotein E, *MMSE* Mini-Mental State Examination, *WMH* white matter hyperintensities

**Table 2:** Factors contributing to tau PET positivity in Aβ+ AD dementia Aβ-positive AD dementia

Aβ-positive AD dementia													
	*N*	Entorhinal cortex				Temporal meta-ROI				Braak V-VI ROI			
		TAU−	TAU+	OR (95% CI)	*P*	TAU−	TAU+	OR (95% CI)	*P*	TAU−	TAU+	OR (95% CI)	*P*
A. Bivariate model													
Age	430	74.6 ± 8.8	70.9 ± 8.8	0.95 (0.92–0.98)	< 0.001	77.3 ± 7.1	70.7 ± 8.8	0.90 (0.86–0.94)	< 0.001	76.5 ± 7.0	68.5 ± 8.9	0.88 (0.85–0.90)	< 0.001
Sex, % male	430	45.1	57.5	1.65 (1.00–2.78)	0.053	49.0	56.4	1.35 (0.74–2.46)	0.324	56.2	49.4	0.96 (0.65–1.42)	0.848
*APOE* ε4+,%	391	59.4	66.6	1.44 (0.85–2.43)	0.174	48.9	67.6	2.21 (1.21–4.11)	0.011	68.5	63.6	0.89 (0.59–1.33)	0.564
MMSE	421	22.5 ± 3.7	20.5 ± 4.6	0.89 (0.82–0.95)	< 0.001	23.5 ± 2.9	20.5 ± 4.6	0.80 (0.73–0.88)	< 0.001	22.4 ± 3.5	19.9 ± 4.8	0.86 (0.81–0.91)	< 0.001
Thickness	422	2.44 ± 0.2	2.41 ± 0.2	0.91 (0.79–1.05)	0.195	2.47 ± 0.2	2.41 ± 0.2	0.91 (0.79–1.05)	0.195	2.43 ± 0.2	2.4 ± 0.2	0.91 (0.79–1.05)	0.195
WMH	303	1.02 ± 1.1	0.88 ± 1.0	0.99 (0.96–1.02)	0.386	1.12 ± 1.0	0.87 ± 1.0	0.98 (0.95–1.01)	0.165	1.12 ± 1.0	0.83 ± 1.0	0.98 (0.96–1.01)	0.131
B. Multivariable model (*n* = 381)													
Age				0.92 (0.88–0.95)	< 0.001			0.86 (0.81–0.91)	< 0.001			0.86 (0.83–0.89)	< 0.001
Sex, % male				1.76 (0.97–3.19)	0.061			1.96 (0.94–4.15)	0.074			1.19 (0.73–1.94)	0.495
*APOE* ε4+,%				1.44 (0.81–2.54)	0.208			2.68 (1.35–5.44)	0.005			0.95 (0.58–1.54)	0.836
MMSE				0.89 (0.81–0.96)	0.006			0.76 (0.66–0.88)	< 0.001			0.86 (0.80–0.92)	< 0.001
Thickness				0.88 (0.74–1.05)	0.157			0.77 (0.62–0.96)	0.023			0.84 (0.73–0.96)	0.016


Reported odds ratios, 95% confidence intervals, and *P* values were derived from bivariate (A) and multivariable (B) binary logistic regression models. AD-signature cortical thickness (*10) and WMH volumes were divided by 10 and 1000, respectively, to obtain odds ratios in a similar range as the other variables. The multivariable model included only participants with all variables available. WMH volumes were excluded from the multivariable models due to missing data

*AD* Alzheimer’s disease, *APOE* apolipoprotein E, *MMSE* Mini-Mental State Examination, *ROI* region of interest, *WMH* white matter hyperintensities

**Table 3:** Factors contributing to tau PET positivity in Aβ+ mild cognitive impairment Aβ-positive mild cognitive impairment

Aβ-positive mild cognitive impairment													
	*N*	Entorhinal cortex				Temporal meta-ROI				Braak V-VI ROI			
		TAU−	TAU+	OR (95% CI)	*P*	TAU−	TAU+	OR (95% CI)	*P*	TAU−	TAU+	OR (95% CI)	*P*
A. Bivariate model													
Age	381	71.7 ± 7.8	71.5 ± 8.3	0.99 (0.97–1.02)	0.764	71.4 ± 7.8	71.8 ± 8.4	1.01 (0.98–1.03)	0.649	72.6 ± 7.5	68.2 ± 8.9	0.93 (0.91–0.96)	< 0.001
Sex, % male	381	38.5	54.5	1.03 (0.58–1.83)	0.002	40.7	53.1	1.65 (1.10–2.48)	0.016	45.6	49.4	1.16 (0.72–1.89)	0.536
*APOE* ε4+,%	337	49.8	74.7	2.49 (1.64–3.83)	< 0.001	55.0	70.0	1.81 (1.21–2.75)	0.005	60.5	67.3	1.45 (0.89–2.38)	0.134
MMSE	374	27.5 ± 2.2	26.5 ± 2.5	0.83 (0.76–0.91)	< 0.001	27.4 ± 2.2	26.6 ± 2.6	0.87 (0.79–0.95)	0.002	27.2 ± 2.3	26.4 ± 2.7	0.88 (0.79–0.97)	0.009
Thickness	367	2.64 ± 0.2	2.56 ± 0.2	0.80 (0.72–0.90)	< 0.001	2.66 ± 0.2	2.54 ± 0.2	0.70 (0.62–0.79)	< 0.001	2.62 ± 0.2	2.56 ± 0.2	0.88 (0.77–0.99)	0.033
WMH	242	0.97 ± 1.0	0.73 ± 0.8	0.97 (0.94–1.00)	0.045	0.91 ± 1.0	0.77 ± 0.8	0.98 (0.96–1.01)	0.248	0.89 ± 0.9	0.65 ± 0.9	0.97 (0.92–1.00)	0.103
B. Multivariable model (*n* = 328)													
Age				0.98 (0.95–1.02)	0.319			0.98 (0.95–1.02)	0.124			0.91 (0.87–0.95)	< 0.001
Sex, % male				1.54 (0.96–2.46)	0.074			1.54 (0.96–2.46)	0.144			0.93 (0.53–1.63)	0.792
*APOE* ε4+,%				2.60 (1.67–4.10)	< 0.001			2.60 (1.67–4.10)	0.004			1.54 (0.91–2.66)	0.112
MMSE				0.86 (0.77–0.95)	0.003			0.86 (0.77–0.95)	0.045			0.86 (0.77–0.97)	0.012
Thickness				0.79 (0.69–0.91)	0.001			0.79 (0.69–0.91)	< 0.001			0.76 (0.65–0.89)	< 0.001


Reported odds ratios, 95% confidence intervals, and *P* values were derived from bivariate (A) and multivariable (B) binary logistic regression models. AD-signature cortical thickness (*10) and WMH volumes were divided by 10 and 1000, respectively, to obtain odds ratios in a similar range as the other variables. The multivariable model included only participants with all variables available. WMH volumes were excluded from the multivariable models due to missing data

*APOE* apolipoprotein E, *MCI* mild cognitive impairment, *MMSE* Mini-Mental State Examination, *ROI* region of interest, *WMH* white matter hyperintensities

**Table 4:** Factors contributing to tau positivity in non-AD neurodegenerative disorders Non-AD neurodegenerative disorders

Non-AD neurodegenerative disorders													
	*N*	Entorhinal cortex				Temporal meta-ROI				Braak V-VI ROI			
		TAU−	TAU+	OR (95% CI)	*P*	TAU−	TAU+	OR (95% CI)	*P*	TAU−	TAU+	OR (95% CI)	*P*
A. Bivariate model													
Age	370	68.9 ± 7.9	73.0 ± 8.7	1.07 (1.03–1.12)	0.002	69.9 ± 8.1	73.3 ± 7.3	1.08 (1.03–1.13)	0.003	69.3 ± 8.1	72.6 ± 8.2	1.07 (1.00–1.14)	0.148
Sex, % male	370	51.8	65.9	1.79 (0.94–3.56)	0.082	52.5	62.9	1.53 (0.75–3.21)	0.247	53.2	61.5	1.41 (0.46–4.73)	0.557
*APOE*ε4+, %	305	28.6	59.0	3.59 (1.81–7.29)	< 0.001	29.7	56.2	3.05 (1.45–6.52)	0.003	32.4	33.3	1.04 (0.27–3.40)	0.947
Aβ+, %	324	21.3	81.0	15.7 (7.2–38.2)	< 0.001	23.2	87.0	17.2 (6.9–52.4)	< 0.001	26.4	92.3	33.5 (6.5–615.3)	< 0.001
MMSE	333	24.9 ± 5.1	19.9 ± 6.2	0.88 (0.83–0.92)	< 0.001	24.7 ± 5.2	20.0 ± 6.2	0.89 (0.84–0.94)	< 0.001	24.4 ± 5.3	19.2 ± 7.3	0.89 (0.83–0.96)	0.003
Thickness	370	2.60 ± 0.2	2.36 ± 0.2	0.68 (0.59–0.77)	< 0.001	2.59 ± 0.2	2.37 ± 0.2	0.03 (0.01–0.05)	< 0.001	2.58 ± 0.2	2.35 ± 0.1	0.72 (0.60–0.88)	< 0.001
WMH	368	0.62 ± 0.8	1.38 ± 1.9	1.05 (1.03–1.08)	< 0.001	0.65 ± 0.8	1.28 ± 1.9	1.04 (1.01–1.07)	< 0.001	0.67 ± 0.8	1.81 ± 2.8	1.05 (1.01–1.08)	0.002
B. Multivariable model (*n* = 284)													
Age				1.03 (0.97–1.10)	0.230			1.02 (0.96–1.10)	0.492			0.93 (0.83–1.03)	0.156
Sex, % male				2.12 (0.89–5.32)	0.097			1.97 (0.77–5.36)	0.166			1.83 (0.83–1.03)	0.417
*APOE*ε4+, %				1.09 (0.45–2.67)	0.841			1.13 (0.43–2.95)	0.806			0.27 (0.06–1.05)	0.067
Aβ+, %				16.0 (5.4–60.0)	< 0.001			14.3 (4.2–66.4)	< 0.001			NA^#^	NA^#^
MMSE				0.91 (0.84–0.98)	0.021			0.91 (0.84–0.99)	0.026			0.92 (0.82–1.03)	0.141
Thickness				0.86 (0.71–1.04)	0.109			0.90 (0.74–1.12)	0.323			0.87 (0.66–1.15)	0.304


Reported odds ratios, 95% confidence intervals, and *P* values were derived from bivariate (A) and multivariable (B) binary logistic regression models. AD-signature cortical thickness (*10) and WMH volumes were divided by 10 and 1000, respectively, to obtain odds ratios in a similar range as the other variables. The multivariable model included only participants with all variables available. WMH volumes were excluded from the multivariable models due to missing data

# 12/13 tau PET–positive cases in the Braak V/VI ROI were Aβ+ (statistical model could not provide an output for this variable)

*AD* Alzheimer’s disease, *APOE* apolipoprotein E, *MMSE* Mini-Mental State Examination, *ROI* region of interest, *WMH* white matter hyperintensities

**Table 5:** Factors contributing to tau positivity in cognitively unimpaired persons Cognitively unimpaired individuals

Cognitively unimpaired individuals													
	*N*	Entorhinal cortex				Temporal meta-ROI				Braak V-VI ROI			
		TAU−	TAU+	OR (95% CI)	*P*	TAU−	TAU+	OR (95% CI)	*P*	TAU−	TAU+	OR (95% CI)	*P*
A. Bivariate model													
Age	1156	67.5 ± 12	73.6 ± 7.0	1.07 (1.04–1.10)	< 0.001	67.5 ± 12	73.3 ± 5.9	1.06 (1.03–1.09)	< 0.001	67.8 ± 12	71.3 ± 11	1.03 (0.99–1.08)	0.137
Sex, % male	1157	55.2	56.6	1.06 (0.66–1.70)	0.819	54.7	64.8	1.52 (0.93–2.55)	0.110	54.9	73.1	2.23 (0.97–5.75)	0.072
*APOE*ε4+, %	917	33.7	60.6	2.95 (1.79–4.94)	< 0.001	34.3	55.2	2.31 (1.36–3.95)	0.002	35.6	54.2	2.14 (0.95–4.86)	0.063
Aβ+, %	1151	31.3	82.2	10.2 (5.7–19.7)	< 0.001	31.3	84.1	11.7 (6.3–23.7)	< 0.001	33.8	57.7	2.67 (1.22–6.02)	0.014
MMSE	1144	29.0 ± 1.2	28.4 ± 1.5	0.72 (0.62–0.85)	< 0.001	29.0 ± 1.2	28.7 ± 1.3	0.87 (0.73–1.04)	0.107	29.0 ± 1.2	28.4 ± 1.9	0.76 (0.60–0.99)	0.033
Thickness	1074	2.75 ± 0.1	2.65 ± 0.2	0.67 (0.57–0.77)	< 0.001	2.75 ± 0.1	2.66 ± 0.2	0.68 (0.58–0.80)	< 0.001	2.74 ± 0.1	2.66 ± 0.2	0.72 (0.57–0.94)	0.011
WMH	747	0.51 ± 0.7	0.95 ± 1.5	1.04 (1.02–1.07)	0.001	0.51 ± 0.7	0.94 ± 1.0	1.04 (1.01–1.07)	0.005	0.54 ± 0.8	0.39 ± 0.5	0.96 (0.77–1.05)	0.569
B. Multivariable model (*n* = 833)													
Age				1.04 (1.01–1.09)	0.030			1.03 (1.00–1.09)	0.090			1.01 (0.96–1.06)	0.739
Sex, % male				1.42 (0.79–2.59)	0.240			2.47 (1.30–4.90)	0.007			3.57 (1.29–11.3)	0.025
*APOE*ε4+, %				1.98 (1.09–3.66)	0.027			1.49 (0.79–2.84)	0.214			2.19 (0.86–5.74)	0.100
Aβ+, %				7.3 (3.6–16.8)	< 0.001			9.3 (4.2–23.9)	< 0.001			1.10 (0.40–3.01)	0.845
MMSE				0.82 (0.67–1.00)	0.046			0.99 (0.79–1.26)	0.900			0.89 (0.66–1.27)	0.482
Thickness				0.81 (0.67–1.00)	0.047			0.84 (0.68–1.04)	0.101			0.80 (0.59–1.09)	0.142


Reported odds ratios, 95% confidence intervals, and *P* values were derived from bivariate (A) and multivariable (B) binary logistic regression models. AD-signature cortical thickness (*10) and WMH volumes were divided by 10 and 1000, respectively, to obtain odds ratios in a similar range as the other variables. The multivariable model included only participants with all variables available. WMH volumes were excluded from the multivariable models due to missing data

*APOE* apolipoprotein E, *MMSE* Mini-Mental State Examination, *ROI* region of interest, *WMH* white matter hyperintensities

## Data Availability

Anonymized data from BioFINDER will be shared by request from a qualified academic investigator for the sole purpose of replicating procedures and results presented in the article and as long as data transfer is in agreement with EU legislation on the general data protection regulation and decisions by the Ethical Review Board of Sweden and Region Skåne, which should be regulated in a material transfer agreement.
